# Do patients with non-traumatic rotator cuff tears differ from healthy individuals in forward head posture, rounded shoulder posture, and thoracic kyphosis?

**DOI:** 10.3389/fmed.2026.1928640

**Published:** 2026-09-17

**Authors:** Kanglin Li, Wenfeng Su, Yufeng Wu, Yibei Zhi, Shihua Gao, Yongdong Jiang

**Affiliations:** 1 Zhongshan Hospital of Traditional Chinese Medicine Affiliated to Guangzhou University of Chinese Medicine, Zhongshan, Guangdong, China; 2 Third Orthopedics Department, Zhongshan City Traditional Chinese Medicine Hospital, Zhongshan, Guangdong, China; 3 Tanzhou People’s Hospital of Zhongshan, Zhongshan City Hospital of Integration of TCM & Western Medicine, Zhongshan, Guangdong, China

**Keywords:** craniovertebral angle, non-traumatic rotator cuff tear, rounded shoulder angle, static posture, thoracic kyphosis angle

## Abstract

**Background:**

Altered cervicothoracic and scapular posture may influence shoulder mechanics, but it remains unclear whether patients with non-traumatic rotator cuff tears show consistent static postural changes. This study compared the craniovertebral angle, rounded shoulder angle, and thoracic kyphosis angle between patients with non-traumatic rotator cuff tears and healthy individuals.

**Methods:**

This single-center, cross-sectional observational study included 39 patients with imaging-confirmed, full-thickness, non-massive, non-traumatic rotator cuff tears scheduled for repair and 30 healthy controls. Thoracic kyphosis angle was measured using a digital gravity inclinometer, while craniovertebral angle and rounded shoulder angle were assessed using photogrammetry. Unadjusted between-group comparisons were performed using variance-robust tests as appropriate. Multivariable linear regression with HC3 heteroscedasticity-robust standard errors was treated as the principal inferential analysis and adjusted for age, body mass index, sex, and manual labor status.

**Results:**

Thoracic kyphosis angle was higher in the case group than in the control group (43.80° vs. 40.23°; mean difference = 3.56°; 95% CI, 0.87–6.26; Cohen's *d* = 0.59; *P* = 0.011). No statistically significant unadjusted between-group differences were identified for rounded shoulder angle (50.41° vs. 48.88°; mean difference = 1.53°; 95% CI, −2.58 to 5.64; Cohen's *d* = 0.19; *P* = 0.458) or craniovertebral angle (50.78° vs. 47.97°; mean difference = 2.81°; 95% CI, −0.70 to 6.33; Cohen's *d* = 0.40; *P* = 0.115). After adjustment, case status remained associated with greater thoracic kyphosis angle (*B* = 3.98°; 95% CI, 1.30–6.66; *P* = 0.004), whereas the adjusted associations with craniovertebral angle and rounded shoulder angle were not statistically significant.

**Conclusion:**

Patients with non-traumatic, full-thickness, non-massive rotator cuff tears had greater thoracic kyphosis than healthy controls. However, the clinical importance of the approximately 3.6° unadjusted difference remains uncertain because protocol-specific measurement error could not be quantified. No statistically significant adjusted associations were identified for craniovertebral angle or rounded shoulder angle; these null findings should be interpreted cautiously given the study's limited sensitivity to moderate or smaller effects.

## 1 Background

Shoulder pain is a prevalent clinical problem that can substantially affect daily activities, work, and upper-limb function. Rotator cuff tears are common in middle-aged and older adults and represent an important source of shoulder pain and functional limitation (1, 2), their prevalence increases with age (3). Understanding non-traumatic rotator cuff tears from a broader biomechanical perspective may therefore be clinically relevant. Although rotator cuff tears primarily involve tendon pathology, shoulder function depends on coordinated motion of the thoracic spine, scapulothoracic articulation, and glenohumeral joint (4), Thoracic posture and scapular position or motion may influence shoulder mechanics, scapulohumeral rhythm, subacromial relationships, and rotator cuff loading (5–8). Experimental studies have also shown that slouched thoracic posture can reduce shoulder range of motion and strength and alter scapular kinematics (7, 8). Because CVA, RSA, and TKA reflect complementary cervical, shoulder-girdle, and thoracic components of posture, examining them together may help determine whether patients with non-traumatic rotator cuff tears demonstrate a broader static postural pattern in addition to local tendon pathology.

Clinically, forward head posture, rounded shoulders, and thoracic kyphosis have been associated with shoulder symptoms (9). The craniovertebral angle (CVA) measures forward head posture, the rounded shoulder angle (RSA) assesses shoulder girdle displacement, and the thoracic kyphosis angle (TKA) quantifies thoracic curvature. Forward head and rounded shoulder postures may influence scapular kinematics and muscle activity, while thoracic alignment may affect shoulder motion and load distribution (10). However, the available evidence is inconsistent. Forward head posture, rounded shoulders, and thoracic kyphosis may occur independently or in different combinations (11) and evidence linking thoracic posture with shoulder pain, range of motion, and function is heterogeneous (12). Scapular kinematics also show substantial inter-individual variability in rotator cuff-related shoulder pain (13). Static postural measures may additionally be influenced by habitual and occupational exposures; recent studies in driver populations have demonstrated differences in CVA and thoracic alignment across driving contexts (14, 15). These findings support interpreting static posture as contextual rather than disease-specific.

Most previous research has focused on subacromial impingement syndrome (16) or frozen shoulder (17), with limited evidence specifically addressing non-traumatic rotator cuff tears. Existing studies have generally examined individual postural parameters or scapular kinematics rather than CVA, RSA, and TKA together. Direct evidence comparing these three static postural measures between patients with imaging-confirmed non-traumatic rotator cuff tears and healthy individuals remains limited.

This study aimed to compare CVA, RSA, and TKA between patients with non-traumatic rotator cuff tears scheduled for repair and healthy controls and to determine whether group status was independently associated with each postural parameter after adjustment for potential confounders. Based on the biomechanical rationale described above, we hypothesized that patients with non-traumatic rotator cuff tears would exhibit a lower CVA, a higher RSA, and a higher TKA than healthy controls.

## 2 Methods

### 2.1 Study design and ethical approval

This single-center, cross-sectional observational study was approved by the Zhongshan Hospital of Traditional Chinese Medicine's Ethics Committee (No. 2025ZSZY-LL-KY-001). All participants provided written informed consent prior to enrollment.

A sample size calculation using G*Power was based on a previous study reporting a mean TKA difference of 4.22°and a standard deviation of 4.83° in a shoulder-disorder population (17), corresponding to Cohen's *d* = 0.87. For a two-tailed test with *α* = 0.05, 90% power, and equal group allocation, an independent-samples *t* test required at least 58 participants, with 29 participants per group.

### 2.2 Participants

Participants were recruited between January 17, 2025, and February 16, 2026. Patients with non-traumatic rotator cuff tears were recruited from the Third Orthopaedics Department of Zhongshan Hospital of Traditional Chinese Medicine, whereas healthy controls were recruited concurrently from individuals undergoing routine health examinations at the same hospital. A total of 41 patients and 35 potential healthy controls were initially assessed. Two patients were excluded after intraoperative assessment demonstrated massive, irreparable rotator cuff tears, and five potential controls were excluded because their BMI was ≥35 kg/m^2^, exceeding the prespecified eligibility threshold. Ultimately, 39 patients and 30 healthy controls were included in the final analysis. Recruitment ended after the minimum sample-size requirement had been exceeded. All participants provided written informed consent. Participant flow is summarized in Supplementary Figure S1.

#### 2.2.1 Inclusion criteria

Participants in the case group were required to satisfy the following criteria:

1. age ≥18 years;
2. present with shoulder symptoms or functional limitations prompting orthopedic evaluation, such as shoulder pain, restricted arm elevation, or reduced shoulder function;
3. have no definitive history of trauma to the affected shoulder, suggesting a non-traumatic onset of the current rotator cuff tear;
4. have a rotator cuff tear confirmed through shoulder magnetic resonance imaging;
5. possess a full-thickness, non-massive, repairable rotator cuff tear. A tear was classified as non-massive and repairable when MRI and, where applicable, intraoperative assessment showed that tendon retraction did not extend medial to the glenoid rim in the coronal or axial plane, tear involvement at the greater tuberosity footprint did not exceed approximately two-thirds (67%) in the sagittal plane, and conventional surgical repair remained feasible;
6. be scheduled for rotator cuff repair following clinical evaluation;
7. have the ability to stand independently and complete assessments of CVA, RSA, and TKA.

Participants in the control group were required to meet the following criteria:

1. age ≥18 years;
2. maintain a healthy status;
3. have no prior history of upper-limb disease;
4. have no history of shoulder pain, shoulder dysfunction, or related medical consultation within the past year;
5. have no known history of structural shoulder disease or spine-related disease;
6. have the ability to stand independently and complete assessments of CVA, RSA, and TKA.

#### 2.2.2 General exclusion criteria

Participants from either group were excluded if they met any of the following criteria:

1. presence of concomitant cervical, thoracic, or lumbar spine pathology;
2. a documented history of shoulder trauma, including, but not limited to, shoulder fracture, recurrent dislocation, or chronic tendon rupture;
3. presence of inflammatory or degenerative shoulder conditions, such as rheumatoid arthritis or osteoarthritis;
4. history of surgical intervention related to the shoulder or spine;
5. diagnosis of neurological disorders, severe musculoskeletal conditions, or any other condition that could potentially influence natural standing posture or affect measurement outcomes;
6. inability to complete postural assessments due to severe pain, restricted positioning, lack of cooperation, or other specific reasons;
7. absence of clinical or measurement data;
8. BMI ≥35 kg/m^2^.

### 2.3 Outcome measures

#### 2.3.1 Demographic and clinical characteristics

Data on age, height, weight, BMI, occupation, manual labor status, dominant arm, CVA, RSA, and TKA were collected for both groups. TKA was the primary outcome, with RSA and CVA as secondary outcomes. The case group also had descriptive clinical information recorded, including affected side, acromial morphology, and subacromial distance. Detailed tear size, individual tendon involvement, symptom duration, pain severity, and standardized shoulder-specific functional scores were not consistently available for all cases and were therefore not included in the analysis.

#### 2.3.2 Measurement procedures

##### 2.3.2.1 Thoracic kyphosis angle

The TKA was assessed utilizing a validated and reliable digital gravity inclinometer (18, 19) (Syntek, Germany). Prior to the formal measurement, participants were instructed to adopt a relaxed and natural standing posture. Foot angle and inter-foot distance were not rigidly prescribed; participants used a comfortable, self-selected stance, and the same standardized natural-standing instructions were repeated before each measurement. The standardized positioning protocol was as follows: Initially, participants were instructed to gently swing their arms forward and backward three times at their sides, subsequently allowing their arms to rest in a position that felt natural and comfortable. Following this, participants were asked to gently flex and extend their head three times before maintaining a natural and comfortable head position. Lastly, participants were directed to take three deep breaths, stabilizing their body posture after the final breath. This procedure was implemented to ensure that participants stood naturally without intentional postural adjustments. The same positioning instructions were reiterated before each data collection session to mitigate postural bias due to instructional discrepancies, which could otherwise affect the measurement outcomes.

Participants were instructed to remove their upper-body clothing to allow full exposure of the spine, while female participants retained close-fitting underwear. A trained examiner, proficient in spinal anatomy and surface landmark identification, identified and marked the spinous processes of the seventh cervical vertebra (C7), the first thoracic vertebra (T1), the twelfth thoracic vertebra (T12), and the first lumbar vertebra (L1). Medical adhesive tape was utilized to demarcate the C7–T1 and T12–L1 positions. The inclinometer was activated and calibrated horizontally in absolute measurement mode. It was initially positioned firmly against the marked C7–T1 location, and the reading was documented. The device was then switched to relative measurement mode, using C7–T1 as the reference horizontal plane, with the angle set to 0°. Subsequently, the inclinometer was positioned firmly against the marked T12–L1 location, and the reading was recorded as the TKA. The skin markings were then removed. Following a 5-minute rest interval, the examiner re-identified the spinous processes and repeated the measurement procedure. The mean value of the two measurements was used as the final result. The measurement procedure is shown in Figure 1.

**Figure 1:**
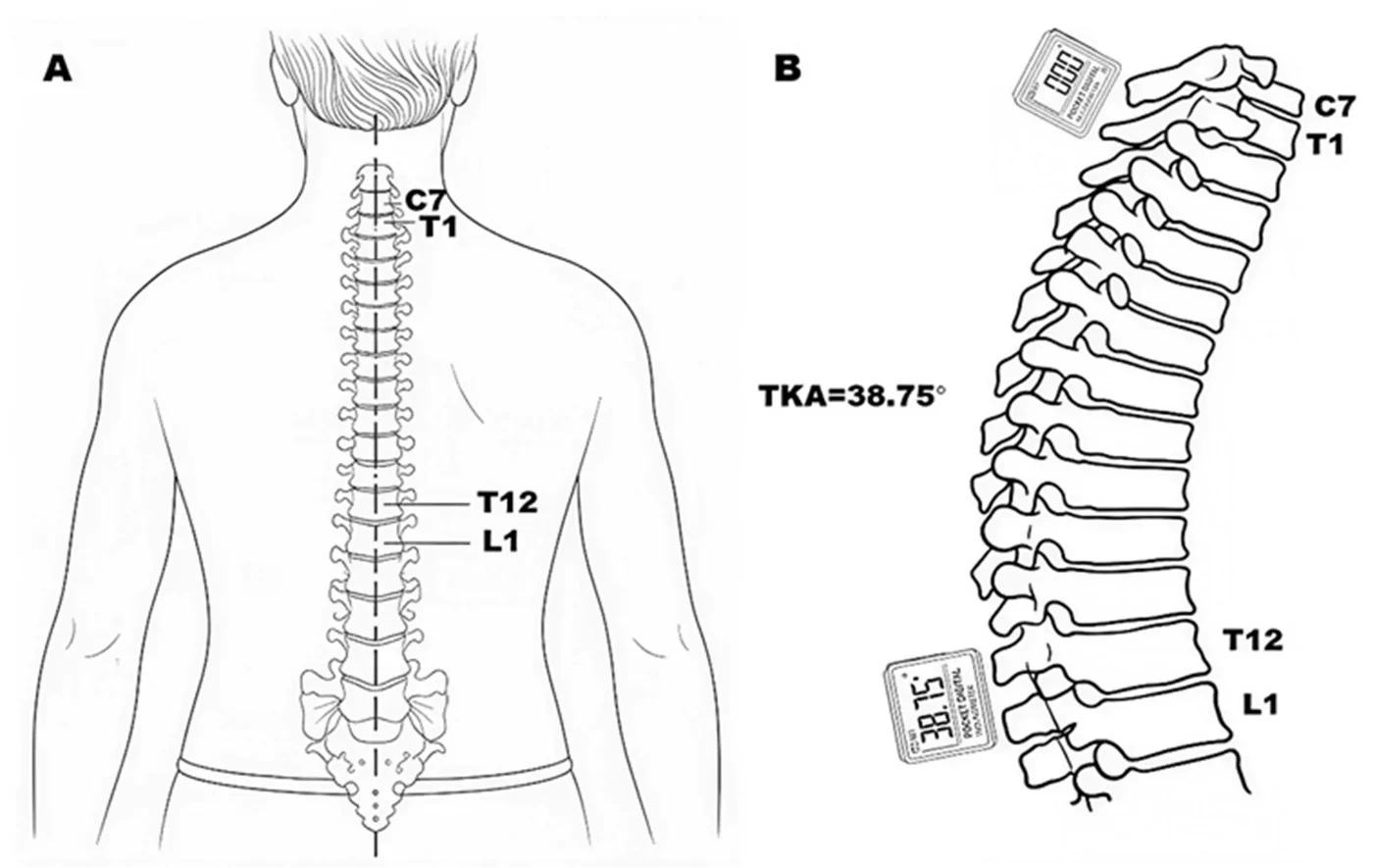
Schematic illustration of TKA measurement using a digital gravity inclinometer.

##### 2.3.2.2 Craniovertebral angle and rounded shoulder angle

CVA is a commonly used photogrammetric measure of forward head posture and is defined as the angle between the line from the C7 spinous process to the tragus and a horizontal line through C7 (20). A CVA <50° has commonly been used as an operational criterion for forward head posture in previous studies, with smaller angles indicating greater forward head posture (21).

Rounded shoulder posture involves anterior curvature of the shoulders, creating a rounded appearance from a side view. The RSA measures this posture by the angle between the line from the acromion's lateral border to the C7 spinous process and a line perpendicular to the ground (22). Normally, this angle is small, but it increases with rounded shoulder posture.

CVA and RSA were assessed using a validated photogrammetric approach (10). After TKA measurement, participants maintained a relaxed standing posture using the same comfortable, self-selected stance, and the tragus, C7 spinous process, and lateral border of the acromion were marked as anatomical landmarks. For patients, lateral photographs were obtained from the affected side. For healthy controls, the measurement side was assigned to approximate the right-to-left affected-side distribution of the case group. Photographs were obtained using an Apple iPhone 13 positioned approximately 2 m from the participant. The native camera application was used with its default settings; focal length was not manually fixed or recorded during data collection, and no additional digital distortion correction was applied before analysis. Blinding during participant positioning and image acquisition was not feasible because of the clinical recruitment setting. However, all photographs were anonymized and coded before analysis, and group allocation was concealed from the assessor performing the Kinovea measurements. Images were analyzed using Kinovea software (version 2025.1.1). For CVA, the angle was constructed between the C7-to-tragus line and a horizontal reference line through C7. For RSA, the angle was constructed between the C7-to-acromion line and a vertical reference line perpendicular to the image horizontal. Each measurement was performed twice by the same examiner, and the mean of the two measurements was used for analysis. The measurement procedure is shown in Figure 2.

**Figure 2:**
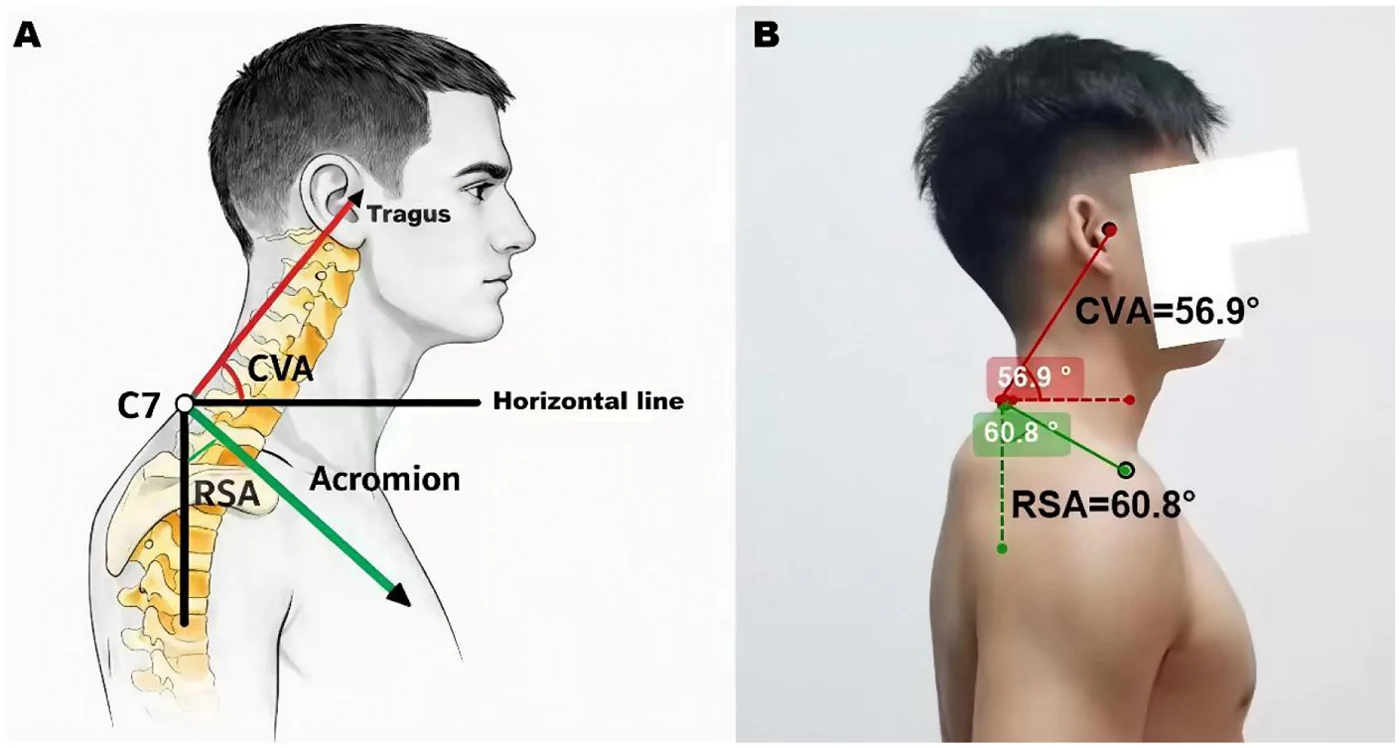
Schematic illustration of CVA and RSA assessment using photogrammetry.

### 2.4 Statistical analysis

Statistical analyses were conducted using R software (version 4.3.3). Normality of continuous variables was assessed using the Shapiro–Wilk test together with visual inspection of Q–Q plots. Normally distributed variables were summarized as mean ± standard deviation, whereas non-normally distributed variables were summarized as median (interquartile range). Homogeneity of variance was evaluated using Levene's test. Welch's *t* test was used for normally distributed continuous between-group comparisons, and the Mann–Whitney *U* test was used for non-normally distributed variables. Categorical variables were compared using the chi-square test or Fisher's exact test, as appropriate. Mean differences, 95% confidence intervals, and Cohen's *d* were calculated for CVA, RSA, and TKA.

Separate univariable and multivariable linear regression models were constructed for CVA, RSA, and TKA, with group status, age, BMI, sex, and manual labor status included as predictors. Model assumptions were evaluated using residual-vs.-fitted plots and the Ramsey RESET test for functional form, Q–Q plots and Shapiro–Wilk tests for residual distribution, variance inflation factors for multicollinearity, the Breusch–Pagan test for heteroscedasticity, and Cook's distance for influential observations. HC3 heteroscedasticity-robust standard errors were used for regression inference. Results are presented as unstandardized coefficients (B), 95% confidence intervals, and *P* values, with statistical significance defined as a two-sided *P* < 0.05.

## 3 Results

### 3.1 Overall comparison

The study included 69 participants: 39 patients with non-traumatic, full-thickness, non-massive rotator cuff tears and 30 healthy controls. Age, sex, height, age category, BMI category, manual labor status, and dominant arm distribution did not differ significantly between groups (all *P* > 0.05). The case group had lower body weight (median [IQR], 60.0 [54.0–67.25] kg vs. 67.75 [60.0–73.0] kg; *P* = 0.004) and lower BMI (23.93 ± 2.58 vs. 26.04 ± 4.28 kg/m^2^; *P* = 0.021). In the case group, the right side was affected in 26 patients (66.67%) and the left side in 13 (33.33%). Acromial morphology was flat in 17 patients (43.59%), curved in 6 (15.38%), and hooked in 16 (41.03%), and the mean subacromial distance was 8.42 ± 1.61 mm (Table 1).

**Table 1:** Baseline and clinical characteristics of the study participants.

Variable	Total (*N* = 69)	Case group (*n* = 39)	Control group (*n* = 30)	Statistic	*P*
Age (years), mean ± SD	58.38 ± 8.72	59.69 ± 8.54	56.67 ± 8.81	Welch *t* = 1.43	0.157
Age category, *n* (%)				*χ*^2^ = 0.05	0.825
≤60 years	45 (65.22%)	25 (64.10%)	20 (66.67%)		
>60 years	24 (34.78%)	14 (35.90%)	10 (33.33%)		
Sex, *n* (%)				*χ*^2^ = 1.38	0.239
Male	29 (42.03%)	14 (35.90%)	15 (50.00%)		
Female	40 (57.97%)	25 (64.10%)	15 (50.00%)		
Height (cm), mean ± SD	160.68 ± 7.70	159.82 ± 7.85	161.80 ± 7.49	Welch *t* = −1.07	0.291
Weight (kg), median (IQR)	65.0 (56.0–70.0)	60.0 (54.0–67.25)	67.75 (60.0–73.0)	*U* = 346.5	0.004
BMI (kg/m^2^), mean ± SD	24.84 ± 3.56	23.93 ± 2.58	26.04 ± 4.28	Welch *t* = −2.39	0.021
BMI category, *n* (%)				*χ*^2^ = 1.16	0.281
≤24 kg/m^2^	35 (50.72%)	22 (56.41%)	13 (43.33%)		
>24 kg/m^2^	34 (49.28%)	17 (43.59%)	17 (56.67%)		
Manual worker, *n* (%)				*χ*^2^ = 0.09	0.765
Yes	40 (57.97%)	22 (56.41%)	18 (60.00%)		
No	29 (42.03%)	17 (43.59%)	12 (40.00%)		
Dominant arm, *n* (%)				Fisher exact	0.379
Right	64 (92.75%)	35 (89.74%)	29 (96.67%)		
Left	5 (7.25%)	4 (10.26%)	1 (3.33%)		
Case-group characteristics					
Affected arm, *n* (%)					
Right	–	26 (66.67%)	–	–	–
Left	–	13 (33.33%)	–	–	–
Acromial morphology (Bigliani), *n* (%)					
Flat	–	17 (43.59%)	–	–	–
Curved	–	6 (15.38%)	–	–	–
Hooked	–	16 (41.03%)	–	–	–
Subacromial distance (mm), mean ± SD	–	8.42 ± 1.61	–	–	–

BMI, body mass index; IQR, interquartile range; SD, standard deviation. Weight was compared using the Mann–Whitney *U* test; other continuous variables were compared using Welch's *t* test. Categorical variables were compared using Pearson's chi-square test or Fisher's exact test, as appropriate.

Shapiro–Wilk testing showed no significant departures from normality for CVA (case: *P* = 0.457; control: *P* = 0.763), RSA (case: *P* = 0.532; control: *P* = 0.627), or TKA (case: *P* = 0.624; control: *P* = 0.082). In the unadjusted comparisons, TKA was higher in the case group than in the control group (43.80° vs. 40.23°; mean difference = 3.56°; 95% CI, 0.87–6.26; Cohen's *d* = 0.59; *P* = 0.011). No statistically significant differences were identified for RSA (50.41° vs. 48.88°; mean difference = 1.53°; 95% CI, −2.58 to 5.64; Cohen's d = 0.19; *P* = 0.458) or CVA (50.78° vs. 47.97°; mean difference = 2.81°; 95% CI, −0.70 to 6.33; Cohen's d = 0.40; *P* = 0.115). Notably, the numerically higher CVA in the case group corresponds to less pronounced forward head posture (Table 2).

**Table 2:** Comparison of postural parameters between groups.

Postural parameter	Total (*N* = 69)	Case group (*n* = 39)	Control group (*n* = 30)	Mean difference (95% CI)	Cohen's *d*	Welch *t*	*P*
CVA (°)	49.56 ± 7.03	50.78 ± 5.95	47.97 ± 8.06	2.81 (−0.70 to 6.33)	0.40	1.60	0.115
RSA (°)	49.75 ± 8.24	50.41 ± 7.61	48.88 ± 9.04	1.53 (−2.58 to 5.64)	0.19	0.75	0.458
TKA (°)	42.25 ± 6.26	43.80 ± 7.46	40.23 ± 3.41	3.56 (0.87 to 6.26)	0.59	2.65	0.011

Data are presented as mean ± SD. CVA, craniovertebral angle; RSA, rounded shoulder angle; TKA, thoracic kyphosis angle; CI, confidence interval. Welch's *t* test was used for all three between-group comparisons.

### 3.2 Univariable and multivariable linear regression analyses

Under HC3 robust inference, group status was not significantly associated with CVA in either the univariable model (*B* = 2.81; 95% CI, −0.74 to 6.37; *P* = 0.119) or the multivariable model (*B* = 2.37; 95% CI, −1.26 to 6.00; *P* = 0.196). Similarly, group status was not significantly associated with RSA in the univariable model (*B* = 1.53; 95% CI, −2.63 to 5.69; *P* = 0.465) or the multivariable model (*B* = 1.97; 95% CI, −2.65 to 6.59; *P* = 0.396). In contrast, case status was associated with greater TKA in both the univariable model (*B* = 3.56; 95% CI, 0.84–6.29; *P* = 0.011) and the multivariable model after adjustment for age, BMI, sex, and manual labor status (*B* = 3.98; 95% CI, 1.30–6.66; *P* = 0.004). BMI was negatively associated with CVA (*B* = −0.50; 95% CI, −0.94 to −0.06; *P* = 0.028) and positively associated with TKA (*B* = 0.33; 95% CI, 0.03–0.64; *P* = 0.032) in the multivariable models (Table 3).

**Table 3:** Univariable and multivariable linear regression analyses of CVA, RSA, and TKA using HC3 robust standard errors.

Outcome	Predictor	Univariable *B* (95% CI)	*P*	Multivariable *B* (95% CI)	*P*
CVA	Group (case vs. control)	2.81 (−0.74 to 6.37)	0.119	2.37 (−1.26 to 6.00)	0.196
	Age	−0.18 (−0.35 to −0.02)	0.031	−0.20 (−0.40 to 0.00)	0.056
	BMI	−0.64 (−1.09 to −0.20)	0.005	−0.50 (−0.94 to −0.06)	0.028
	Sex (male vs. female)	0.89 (−2.67 to 4.45)	0.619	−0.22 (−3.89 to 3.46)	0.907
	Manual worker (yes vs. no)	1.98 (−1.61 to 5.57)	0.274	1.19 (−2.30 to 4.67)	0.499
RSA	Group (case vs. control)	1.53 (−2.63 to 5.69)	0.465	1.97 (−2.65 to 6.59)	0.396
	Age	−0.03 (−0.19 to 0.14)	0.741	−0.07 (−0.27 to 0.14)	0.515
	BMI	0.14 (−0.40 to 0.68)	0.616	0.18 (−0.36 to 0.71)	0.516
	Sex (male vs. female)	−0.67 (−4.62 to 3.28)	0.737	−0.48 (−5.27 to 4.32)	0.843
	Manual worker (yes vs. no)	−2.07 (−6.13 to 1.98)	0.311	−1.85 (−6.18 to 2.47)	0.395
TKA	Group (case vs. control)	3.56 (0.84 to 6.29)	0.011	3.98 (1.30 to 6.66)	0.004
	Age	0.17 (−0.03 to 0.37)	0.100	0.14 (−0.06 to 0.35)	0.174
	BMI	0.11 (−0.22 to 0.44)	0.498	0.33 (0.03 to 0.64)	0.032
	Sex (male vs. female)	−1.01 (−4.15 to 2.12)	0.521	0.33 (−2.82 to 3.48)	0.835
	Manual worker (yes vs. no)	2.09 (−0.98 to 5.16)	0.178	2.83 (−0.24 to 5.90)	0.070

*B*, unstandardized regression coefficient; BMI, body mass index; CI, confidence interval; CVA, craniovertebral angle; RSA, rounded shoulder angle; TKA, thoracic kyphosis angle. Reference categories were the control group for group status, female for sex, and non-manual worker for manual labor status. Age and BMI were modeled as continuous variables. HC3 heteroscedasticity-robust standard errors were used for all regression inference.

Regression diagnostics showed no material multicollinearity (all variance inflation factors <1.17) or evidence of functional-form misspecification. Heteroscedasticity was detected for the TKA model, supporting the use of HC3 robust inference. A sensitivity analysis excluding observations with Cook's distance >4/n did not materially alter the adjusted TKA association (*B* = 3.39; 95% CI, 0.84–5.94; *P* = 0.010).

## 4 Discussion

### 4.1 Main findings

This study compared CVA, RSA, and TKA in patients with non-traumatic, full-thickness, non-massive rotator cuff tears and healthy controls. Among the three postural parameters, TKA showed the clearest between-group association. The unadjusted TKA difference was approximately 3.6° with a moderate effect size, and case status remained associated with greater TKA after adjustment for age, BMI, sex, and manual labor status using HC3 robust inference. No statistically significant adjusted associations were identified for CVA or RSA. BMI was negatively associated with CVA and positively associated with TKA in the robust multivariable models.

The non-significant CVA and RSA findings should not be interpreted as evidence of equivalence. The achieved sample could detect standardized differences of approximately *d* = 0.69 at 80% power, whereas the observed effect sizes were *d* = 0.40 for CVA and *d* = 0.19 for RSA. Accordingly, smaller or moderate between-group effects, particularly for CVA, may have remained undetected.

Despite the prespecified BMI eligibility criterion of <35 kg/m^2^, the control group had a significantly higher mean BMI than the case group. This imbalance was not anticipated and may partly reflect differences between the orthopedic surgical population and the routine health-examination population from which the two groups were recruited. In the revised robust model, BMI was positively associated with TKA (*B* = 0.33; 95% CI, 0.03–0.64; *P* = 0.032), a direction that would tend to predict greater TKA in participants with higher BMI. Nevertheless, the case group showed greater TKA despite its lower BMI, and the adjusted association between case status and TKA persisted. BMI alone therefore does not explain the observed group difference, although residual confounding and differences in the representativeness of the source populations cannot be excluded.

### 4.2 Potential biomechanical link between increased TKA and non-traumatic rotator cuff tears

Greater TKA remained associated with case status after adjustment for measured confounders. This finding suggests a group-level association between thoracic posture and this selected population of patients with non-traumatic rotator cuff tears; however, the cross-sectional design does not establish whether greater thoracic kyphosis preceded the tear, developed in response to shoulder symptoms, or reflects other shared factors.

Arm elevation requires coordinated motion of the thoracic spine, scapulothoracic articulation, and glenohumeral joint. Thoracic posture can influence the distribution of motion across these regions, and restricted thoracic movement may be accompanied by altered scapular kinematics (23, 24). In subacromial impingement syndrome, limited thoracic extension has been associated with changes in scapular tilt, rotation, and protraction (25). Related work in rotator cuff-related shoulder pain also supports considering thoracic, scapular, and glenohumeral mobility as interconnected components of shoulder mechanics (26). These observations provide a plausible biomechanical context for the association observed in the present study but do not establish a causal pathway.

One possible explanation is that a more kyphotic thoracic alignment may reduce available thoracic extension and alter scapular motion during arm elevation. In theory, these changes could modify scapulohumeral rhythm, subacromial relationships, and rotator cuff loading. However, the present study did not measure dynamic scapular kinematics, tendon loading, or longitudinal changes in posture. These mechanisms should therefore be regarded as biomechanical hypotheses rather than causal explanations for rotator cuff tearing.

### 4.3 Interpretation of CVA findings

Forward head posture can theoretically impact cervicothoracic alignment and scapular position by affecting muscles like the upper trapezius and levator scapulae. Thigpen et al. found that this posture changes scapular movement and muscle activity in those without shoulder pain (10). Dehqan et al. noted that severe forward head posture reduces the subacromial space during arm elevation, potentially affecting the rotator cuff (27).

The mean CVA was numerically higher in the case group than in the control group (50.78° vs. 47.97°), which corresponds to less pronounced forward head posture. This difference was not statistically significant in the unadjusted Welch comparison (*P* = 0.115) or in the adjusted robust model (*P* = 0.196). Given the moderate observed effect size (Cohen's *d* = 0.40) and the limited sensitivity of the achieved sample to detect an effect of this magnitude, the CVA finding should be considered inconclusive rather than evidence of equivalence between patients and controls. Habitual and occupational posture may also influence CVA independently of shoulder pathology (14, 15, 28).

BMI remained negatively associated with CVA in the robust multivariable model, whereas the association with age did not reach statistical significance. Previous work has also linked body composition and age-related factors with head posture (28). These findings reinforce the need to consider individual characteristics when interpreting CVA. Recent clinimetric work has shown high reliability for smartphone-based CVA assessment, although measurement properties are specific to the device, software, and protocol used (29).

### 4.4 Possible explanations for the absence of an overall difference in RSA

No statistically significant unadjusted or adjusted association between group status and RSA was identified. Although rounded shoulder posture may theoretically influence scapular alignment, the subacromial space, and rotator cuff loading, the present data do not support RSA as a consistent group-level discriminator between patients with non-traumatic rotator cuff tears and healthy controls.

This lack of a group-level association is compatible with evidence that forward head posture, rounded shoulders, and increased thoracic kyphosis can occur independently or in different combinations (11). Greenfield et al. also reported no clear postural distinction between individuals with shoulder overuse injuries and healthy controls (30).

Current evidence further emphasizes substantial variability in posture and movement patterns in shoulder disorders. Reviews have reported inconsistent relationships between thoracic posture and shoulder pain, motion, and function, as well as marked variability in scapular kinematics among individuals with rotator cuff-related shoulder pain (12, 13). Thus, a plausible scapular mechanism does not necessarily translate into a consistent difference in a single static RSA measurement.

The absence of a statistically significant RSA association in this study may therefore reflect the influence of individual scapular control, thoracic posture, habitual activity, and task demands. Larger studies incorporating dynamic scapular measures may be better suited to determine whether clinically relevant RSA-related subgroups exist.

Importantly, the group means represent continuous postural measurements and should not be interpreted as indicating that all participants met categorical criteria for a postural abnormality. Healthy control status in this study reflected the absence of relevant shoulder and spine disorders rather than a requirement for ideal static postural alignment. Because CVA, RSA, and TKA were study outcomes, participants were not selected or excluded according to these measurements.

### 4.5 Clinical implications and directions for future research

The present study cannot determine whether greater TKA was a pre-existing characteristic or developed in response to chronic shoulder symptoms. Previous research has reported increased thoracic kyphosis in patients after arthroscopic rotator cuff repair compared with healthy individuals (31), supporting continued investigation of the temporal relationship between thoracic posture and rotator cuff disorders.

The clinical relevance of the approximately 3.6° unadjusted TKA difference remains uncertain. Lewis and Valentine reported standard errors of measurement of 1.0° in asymptomatic participants and 1.7° in participants with shoulder symptoms for averaged thoracic kyphosis measurements obtained using gravity-dependent inclinometers (32). Using MDC95 = SEM × 1.96 × √2, these values correspond to approximate MDC95 values of 2.8° and 4.7°, respectively. However, their measurement protocol differed from the present protocol, and these values cannot be directly transferred to our study. Because the individual replicate measurements were not retained separately, a protocol-specific MDC could not be calculated, and it cannot be determined whether the observed TKA difference exceeded the measurement error of the present protocol.

From a hypothesis-generating perspective, TKA may provide supplementary information about the overall postural characteristics of patients with non-traumatic, full-thickness, non-massive rotator cuff tears undergoing repair. However, TKA should not be interpreted as a diagnostic marker or used as a stand-alone basis for treatment decisions. The present cross-sectional findings also do not establish that rehabilitation should be modified according to thoracic posture. Scapular-focused interventions have shown potential benefits in other shoulder pain populations (33–36), but whether thoracic mobility, thoracic extension, or scapular-control interventions provide additional benefit in patients with rotator cuff tears and increased thoracic kyphosis requires prospective and interventional evaluation.

### 4.6 Strengths and limitations

This study has several strengths. It focused on a relatively homogeneous group of patients with imaging-confirmed, non-traumatic, full-thickness, non-massive rotator cuff tears and evaluated three complementary static postural parameters. Effect sizes and confidence intervals were reported, and the principal multivariable analyses adjusted for age, BMI, sex, and manual labor status. Statistical assumptions were re-evaluated, and HC3 robust inference together with influential-observation sensitivity analysis was used to improve the robustness of the main findings.

Several limitations should be considered. First, the cross-sectional design permits assessment of association but not temporality or causality. Static postural measurements do not capture dynamic thoracic or scapular movement. Second, healthy controls did not undergo shoulder MRI; therefore, asymptomatic rotator cuff tears could not be excluded, introducing the possibility of control-group misclassification. Third, detailed tear characteristics, individual tendon involvement, symptom duration, pain severity, and standardized shoulder-specific functional scores were not consistently available for all participants and could not be incorporated into the analysis.

Fourth, although each postural parameter was measured twice and the mean value was used for analysis, the individual replicate measurements were not retained separately in the final analytical dataset. Consequently, study-specific intra-rater reliability, SEM, and MDC could not be calculated retrospectively. In addition, foot angle and inter-foot distance were not rigidly standardized, which may have introduced additional variability in natural standing posture. Fifth, the initial sample-size calculation was based on a frozen-shoulder population and a relatively large anticipated effect size; the achieved sample had limited sensitivity to detect moderate or smaller effects, particularly for CVA and RSA. Sixth, body weight and BMI differed between groups, and the case and control groups arose from different source populations, so residual confounding and selection effects cannot be excluded. Finally, the case group consisted of patients with non-traumatic, full-thickness, non-massive, repairable tears selected for surgical treatment; the findings should not be directly extrapolated to partial-thickness, massive or irreparable, less symptomatic, or conservatively managed rotator cuff tears.

## 5 Conclusion

Among patients with non-traumatic, full-thickness, non-massive rotator cuff tears, case status remained associated with greater TKA after adjustment for age, BMI, sex, and manual labor status, whereas no statistically significant adjusted associations were identified for CVA or RSA. The clinical relevance of the observed TKA difference remains uncertain because protocol-specific measurement error could not be quantified, and the cross-sectional design precludes causal inference. Further prospective studies are needed to clarify the relationship between thoracic posture and non-traumatic rotator cuff tears.

## Data Availability

The raw data supporting the conclusions of this article will be made available by the authors, without undue reservation.
